# Editorial: Tumor-promoting immune cells: Cancer immune escape and beyond

**DOI:** 10.3389/fimmu.2023.1168884

**Published:** 2023-03-03

**Authors:** Charlotte Domblides, Darya Alizadeh, Nicolas Larmonier

**Affiliations:** ^1^ Centre National de la Recherche Scientifique (CNRS) Unité Mixte de Recherche (UMR) 5164, ImmunoConcEpT, University of Bordeaux, Bordeaux, France; ^2^ Department of Medical Oncology, Bordeaux University Hospital, Bordeaux, France; ^3^ Biological and Medical Sciences Department, University of Bordeaux, Bordeaux, France; ^4^ T Cell Therapeutics Research Labs, Cellular Immunotherapy Center, Department of Hematology and Hematopoietic Cell Transplantation, City of Hope, Duarte, CA, United States

**Keywords:** immunosuppression, cancer immune escape, myeloid cells, Treg - regulatory T cell, tumor microenvironment, immunosuppressive immune cells, immunotherapies

Most cancers emerge and develop in a dedicated tumor-promoting environment. Compelling evidence has been provided that immune cells critically contribute to, and shape this tumor landscape. Efficient protective innate and adaptive anti-tumoral immune responses, primarily attributed to diverse immune effector subsets, such as cytotoxic CD8^+^ T lymphocytes (CTL), CD4^+^ helper T lymphocytes (Th), γδ T cells, Natural Killer cells (NK), NKT, dendritic cells or anti-tumoral macrophages, are profoundly altered in this context. Conversely, many different populations of immunosuppressive immune cells play a central role in the mechanisms of cancer escape from anti-tumor immunity. These tumor-induced, tumor-promoting immune subsets include cells of the lymphoid lineage (a large variety of immunosuppressive CD4^+^ and CD8^+^ “regulatory T lymphocytes”, Tregs), and of myeloid origin (primarily tumor-associated macrophages (TAMs), tumor-associated neutrophils (TANs), tolerogenic dendritic cells (tDC) and immature subsets of myeloid cells endowed with immunosuppressive properties that have collectively been termed “myeloid-derived suppressor cells” (MDSCs)). Induced by tumor-derived factors, these cells accumulate in the tumor microenvironment, but also at the sites of priming of anti-tumoral immune responses, in the bloodstream and in the metastatic niches. Increased frequency of these immune cell populations usually (but not always) correlates with a negative prognostic and relapse of many cancer patients. The modalities by which these cells impair the different steps of anti-tumoral immune responses have been extensively deciphered and involve very broad mechanisms, including the production of soluble and/or membrane-bound immunosuppressive factors such as Tumor Growth Factor (TGF)-β, and/or the expression of enzymes involved in amino acid metabolism such as indoleamine 2,3-dioxygénase (IDO) or arginase (Arg).

However, beyond their cardinal immunosuppressive properties, most of these immune lymphoid or myeloid subsets can also exhibit multiple “non-immunological” tumor-promoting functions. Some of these populations can indeed directly enhance tumor cell survival and proliferation, contribute to the epithelial-to-mesenchymal transition (EMT) and to cancer cell stemness, participate to local tissue invasion, foster blood or lymphatic vessel intravasation and extravasation of migrating cancer cells. Additionally, it has been reported that some subsets of myeloid cells can associate with circulating tumor cells, protecting them in the bloodstream and can prepare the pre-metastatic niches thus enhancing malignant cell metastasis. In addition, the contribution of these cells to cancer resistance to chemotherapeutic agents and to endocrine therapies has also been widely described.

As these immunosuppressive T lymphocytes and myeloid cells significantly participate to most key processes responsible for tumor development and dissemination, their therapeutic targeting (elimination, inactivation or reprogramming) has consistently been associated with successful anti-tumor responses. Unfortunately, the extreme phenotypic and functional heterogeneity and the high degree of plasticity of these cells in time and space has prevented their systematic use as reliable biomarkers and represents a current challenge in the development of therapeutic approaches to selectively inhibit their generation, development, and multiple tumor-promoting functions. In this Research Topic of *Frontiers in Immunology*, contributing authors focus on the limits related to current classifications of suppressive myeloid cells based on their phenotype, functions and metabolic characteristics in different cancer types, on the contribution of recent single-cell technics to the identification of these cells and on the role and mechanisms of action and regulation of regulatory T lymphocytes and suppressive myeloid cell subsets in the tumor environment. Therapeutic interventions to overcome the tumor-promoting effects of these immune cells in cancers are further assessed and discussed.

In a systematic review assessing the multifaced tumor-promoting functions of dedicated subpopulations of myeloid cells in breast cancers, Blaye et al. highlight the challenges related to the heterogeneous nature of these cells and the phenotypical and functional overlaps between subsets. Current pitfalls preventing the unequivocal discrimination of distinct subsets of suppressive myeloid cells are discussed. This problem is particularly striking in the case of Polymorphonuclear (PMN)-MDSC and immunosuppressive neutrophils, prompting the authors to propose to globally name these cells IMCGL (immunosuppressive myeloid cells of the granulocytic lineage). The value of individual subsets as biomarkers and potential therapeutic targets, and whether distinct subsets may be endowed with one unique or with multiple specialized tumor-promoting functions, concomitantly or acquired over time remain important issues to be addressed in the future. Along these lines, Larkin et al. address the complexity of the myeloid landscape, a predominant element of glioblastoma (GBM) microenvironment, and particularly emphasize the origins and heterogeneity of macrophage subsets in GBM. The authors extensively analyze and discuss the main breakthroughs made by recent single-cell technologies (specifically scRNA-seq and CyTOF) at the transcriptomic and proteomic levels, towards a deeper and more precise characterization of myeloid cells in GBM. The authors also assess the possibilities to further harness these technologies before and after patient therapy to explore mechanisms of resistance or response to therapies. The limits and future improvements of these approaches are further discussed. Similarly, Lin et al. review the main myeloid elements of the glioma environment including glioma-associated macrophages/microglia, neutrophils, dendritic cells and MDSCs, with a specific emphasis on the cross-talks between these cells and malignant cells, and on their ambivalent roles (pro- *versus* anti-tumoral) in cancer development. Specific targeting of these glioma-associated myeloid cells is considered. In a focused review, Chen et al. provide an extensive description of the characteristics (composition in proteins and RNAs) and impact of exosomes produced by MDSC associated with different tumors on cancer immunity, angiogenesis, metastasis and resistance to therapies. The underlying mechanisms of action of these extracellular vesicles and their potential prognostic and therapeutic values are detailed and discussed. In an effort to further comprehend the implications of MDSC in immune escape mechanisms in melanomas, Marguier et al. extensively studied the characteristic of a novel subset of monocytic MDSC that overexpress the receptor for the pro-angiogenic factor angiopoietin 2 (Tie-2). The authors specifically demonstrate that Tie-2-expressing MDSC exhibiting immunosuppressive features are increased in melanoma patients, and that, interestingly, stimulation of Tie-2 signaling enhances the immunosuppressive functions of these cells. These results thus suggest that targeting Tie-2/angiopoietin axis may offer a potential new therapeutic option to improve immunotherapies. Using melanoma and lung cancer mouse models, Papafragkos et al. highlight the implication of the NLRP3 inflammasome in MDSC function, particularly showing that monocytic and granulocytic MDSC suppressive activity is impaired in NLRP3 KO mice. The authors further provide data suggesting that inhibition of NLRP3 using pharmacologic agents also alter MDSC activities, resulting in reduced tumor development and advocating for a possible interest in NLRP3 targeting to augment the efficacy of immune-based therapies.

Analyzing the lung microenvironment of non-small cell lung cancer patients (NSCLC), Heim et al. detected IL-9 and IL-21 production by both tumor-infiltrating T lymphocytes (TIL) and by malignant cells and demonstrate the presence of FoxP3-expressing Treg producing IL-9 in the lung microenvironment. Assessing the relevance of these findings in mouse lung cancer models, the authors establish that IL-9 deletion or IL-9 blockade using antibodies leads to inhibition of tumor development. Furthermore, IL-9 receptor-expressing tumor cells, TIL and Treg were identified as target of IL-9. These results thus provide further evidence for the role (and thereby potential targeting interest) of IL-9/IL-9 producing cells in the mechanisms of immune escape in NSCLC. In an analysis by immunohistochemistry of tissue microarrays from rectal cancer patients, Schnellhardt et al. described data further supporting the notion that immunosuppressive FoxP3^+^ Treg are not always associated with negative prognostic but may conversely represent positive factors depending on the relative abundance of tumor-infiltrating CD8^+^ T lymphocytes.

In additional articles, Jia et al. review, in the context of primary hepatic carcinoma, the composition, origin, formation, distribution and the controversial prognostic value of tertiary lymphoid structures (TLS), which correspond to ectopic lymphatic edifices containing lymphocytes, myeloid cells, and interstitial cells and involved in tumor immunity, while Wang et al. summarizes the role and modulation of the tumor immune landscape, including suppressive myeloid cells and Treg, in the response or resistance to checkpoint inhibitor anti-PD1 therapies. Yu et al. focus on the possible role and relevance of CD47 in ovarian cancer immune microenvironment and Liu et al. assess the implication of the complement regulatory protein CD55 (DAF, decay acceleration factor) in colon malignancies. Li and Liu discuss the possible prognostic relevance of activator of HSP90 ATPase activity 1 in relation with the tumor immune landscape in different cancer types, and finally, Song et al. review and discuss the regulation and immunosuppressive modes of action of the enzyme IDO1, as one out of many mechanisms of tumor-induced immunosuppression and tumor immune escape.

## Author contributions

All authors have equally contributed to the design, structure, writting and proofreading of the manuscript.

